# Secondary Root Canal Treatment with Reciproc Blue and K-File: Radiographic and ESEM-EDX Analysis of Dentin and Root Canal Filling Remnants

**DOI:** 10.3390/jcm9061902

**Published:** 2020-06-18

**Authors:** Carlo Prati, Fausto Zamparini, Andrea Spinelli, Gian Andrea Pelliccioni, Chiara Pirani, Maria Giovanna Gandolfi

**Affiliations:** 1 Endodontic Clinical Section, School of Dentistry, Department of Biomedical and Neuromotor Sciences (DIBINEM), Alma Mater Studiorum University of Bologna, 40125 Bologna, Italy; fausto.zamparini2@unibo.it (F.Z.); andrea.spinelli4@unibo.it (A.S.); gian.pelliccioni@unibo.it (G.A.P.); chiara.pirani4@unibo.it (C.P.); 2 Laboratory of Green Biomaterials and Oral Pathology, School of Dentistry, Department of Biomedical and Neuromotor Sciences, University of Bologna, 40125 Bologna, Italy; mgiovanna.gandolfi@unibo.it

**Keywords:** retreatment, NiTi instruments, Reciproc Blue, ESEM-EDX, periapical X-Ray, K-file

## Abstract

Secondary root canal treatment requires the complete removal of filling materials with different chemical-physical properties. A newly developed single-use NiTi instrument (Reciproc Blue, RB) may be more effective in root canal retreatment. The aim of the present study was to evaluate morphology and composition of remnants after retreatment with RB compared to traditional K-File technique, in canals obturated with Thermafil/AH Plus. Twenty-four single-rooted human teeth were shaped with NiTi obturated with AH-Plus/Thermafil and retreated using RB NiTi instruments or manual K-Files. Radiographs were taken to evaluate endodontic space and radiopacity of residual filling-material before/after procedures. After retreatment, samples were longitudinally split and observed by environmental scanning electron microscopy connected to energy dispersive X-Ray spectroscopy (ESEM-EDX) to analyze the debris/remnant position, microchemistry, and dentinal surface morphology. Time for retreatments was recorded and compared using one-way ANOVA (*p*-value = 0.05). Radiopaque filling residuals were found in both groups. RB system resulted statistically faster than manual K-File in retreatment procedure (*p* < 0.001). Root canal space radiographic appearance obtained after retreatment with RB was wider than K-File (*p* < 0.05). ESEM-EDX revealed 4 different morphological dentin area. Area-1: debris-free with typical Ca, P, and N composition of dentin and detected in 70% of the surface. Area-2: presence of deproteinized smear layer free from N and debris in 15% of the surface. Area-3: a thick packed smear layer N-free and with fine debris consisting of trace elements from sealer in 10% of the surface. Area-4: packed with debris and trace elements. No difference was observed between both instruments regarding root canal space appearance and ESEM-EDX analysis. Both systems were able to remove filling material but created a dentine morphology composed of packed debris and filling materials embedded into the smear layer. Dentin surface composition resulted in collagen depleted by irrigation procedures. The reciprocating system required less time to complete retreatment.

## 1. Introduction

The main concept behind secondary root canal treatment procedures is the removal of all root canal filling materials infected debris and dentin smear layer that are responsible for periapical disease [1,2,3]. Root canal space is filled with materials with different physical and chemical properties that must be removed. Sealers, gutta-percha points, plastic-based carrier, and other adhesive/composite materials are brittle, with strong consistency and plastic so they can create a great volume of debris inside the root canal during the retreatment procedures.

The five-year success percentage of secondary root canal treatments is lower than primary root canal treatment and ranges approximately 75–80% [4,5] These procedures are often time-consuming and usually require several numbers of sessions, potentially compromising tooth integrity [5]. Traditional instrumentation techniques require the use of a series of manual files or rotary NiTi instruments which may undergo fractures, leading to inadequate root canal treatment. For these issues, several new approaches and techniques are being developed.

New single-file reciprocating NiTi instruments have been introduced for root canal treatment [6] and some attempt to use them for re-treatments has been proposed in vitro [7]. The mechanical properties of Reciproc Blue (RB) suggest a possible use in secondary root canal treatments [8]. Fatigue tests demonstrated that the improving mechanical properties of NiTi instruments are consistent, suggesting their use for secondary root canal treatments [9]. To the best of our knowledge, no study is present in literature analyzing these new NiTi instruments in standard retreatment procedures.

Different in vitro techniques have been proposed to investigate the effectiveness of instruments to remove debris from previously filled root canals, such as micro-CT [10], or scanning electron microscopy (SEM) [11]. Some studies used the environmental scanning electron microscopy (ESEM) to investigate dentin and humid dentin substrates under environmental conditions in situ and in vitro [12]. Energy dispersive X-ray spectroscopy (EDX) may be used in association with ESEM for microchemical analysis of mineralized tissues, such as dentine or bone [13]. Currently, limited information regarding the composition and location of root canal filling debris after secondary root canal treatment is present in literature.

The aim of this study was to simulate in vitro the clinical conditions that characterize secondary root canal treatment of teeth obturated with an epoxy-resin-based sealer (AH Plus) and a gutta-percha plastic-carrier based system and to evaluate the residual radiopacity, the variation of root canal space area measured on radiographic images, and time for retreatment.

ESEM-EDX was used to detect morphology, elemental composition of dentin, and chemical modification induced by irrigation and retreatment instrumentation techniques. EDX spectroscopy was innovatively used to identify and to detect remnants and debris composition still present on dentin surface after retreatment procedures.

## 2. Experimental Section

Twenty-four human teeth extracted for orthodontic or periodontal reasons were stored in a solution of thymol 0.1% and selected by X-ray examination, using the following criteria: single straight canal without root treatment, completely formed apex and absence of canal calcifications. Crowns were removed through a highspeed water-cooled handpiece with a diamond cylindrical bur (Intensiv SA, Montagnola, Swiss), at 13.0 mm from the apex measured with an endodontic measuring gauge (Dentsply Maillefer, Ballaigues, Swiss).

**Figure 1:**
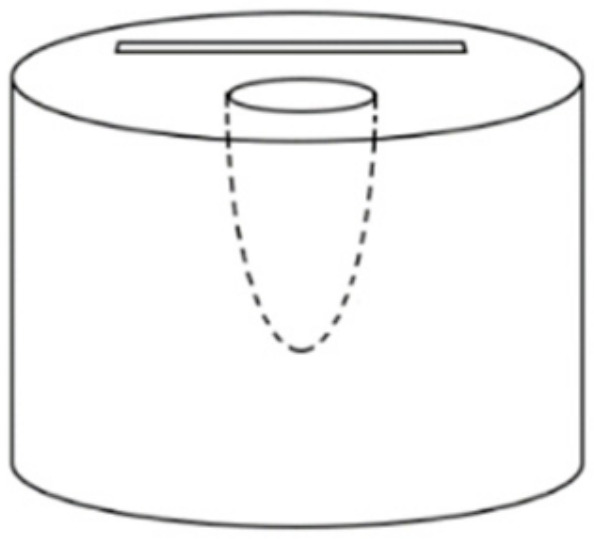
Plastic custom container to fit root sample during treatment and retreatment procedure. The system was used to simulate clinical condition and allow periapical radiographies.

Each root was fixed in a customized plastic container using a polyvinylsiloxane impression material (Optosil base, Kulzer GmbH, Munich, Germany) to clinically simulate the alveolar position and to allow the radiographic survey (Figure 1). All the endodontic procedures were performed with the roots located in this standardized position. A preoperative X-ray of each root was performed. The target-film distance was approximately 30 cm from sample, 0.41 s exposure at 70 kV and 8 mA (MyRay, Cefla, Imola, Italy). X-rays (Kodak Ultraspeed, Carestream, Rochester, NY, USA) were developed in a standard developer unit at 25 °C, (Euronda s.p.a., Vicenza, Italy), 12 s developing and 25 s fixing, according to the manufacturer instructions.

### 2.1. First Root Canal Treatment

A 25mm #10 manual K-File (Dentsply Maillefer, Ballaigues, Swiss) was used to assess root canal patency and to evaluate the working length (WL). Root canals were shaped with HyFlex CM rotary NiTi instruments following the single-length sequence: 25/08 for the coronal third, 20/04–25/04–20/06 (Coltene, Altstätten SG, Switzerland) at WL activated by X-Smart Plus micromotor (Dentsply Maillefer, Ballaigues, Swiss) at 450 RPM, 2.5 Ncm^2^. Each canal was irrigated with 5 mL of 5.25% NaOCl (Niclor 5 Ogna, Muggiò, Italy) and 3 mL of 10% EDTA (Tubuliclean Ogna, Muggiò, Italy) solutions. At the end of instrumentation, each canal was subjected to further irrigation with 1.0 mL of 5.25% NaOCl for 3 min, 0.5 mL 10% EDTA for 1 min and 3 min with 1.0 mL of NaOCl. 

Root canal filling procedures were performed using a carrier-based technique (Thermafil system, Dentsply DeTrey GmbH, Konstanz, Germany) associated with an epoxy-resin based sealer (AH Plus, Dentsply DeTrey GmbH, Konstanz, Germany). AH Plus is a hydrophobic epoxy resin-based sealer containing calcium tungstate (CaWO_4_) and Zirconium Oxide (ZrO_2_). Thermafil obturators are composed of an internal plastic core and coated by a layer of gutta-percha. The inorganic components are mostly Zinc Oxide (ZnO) and radiopacifiers, such as Barium Sulphate (BaSO_4_) or Titanium Dioxide (TiO_2_) [14].

Each canal was dried with sterile paper points and filled with AH Plus and Ø25 Thermafil obturators inserted with a slow and constant movement to the WL. After root canal filling, a periapical radiography was acquired. Samples were then stored in 15 mL Hank’s Balance Salt Solution (HBSS) used as simulated body solution in plastic containers, for 7 days at 37 °C and 95% humidity. All these procedures were performed by two undergraduate students. 

### 2.2. Retreatment Procedure with Reciproc Blue 

All samples were randomly assigned to RB (*n* = 12) or manual K-File retreatment group (*n* = 12). Two trained endodontists performed all the retreatment procedures. An initial pathway was created with Gates-Glidden burs #3–#4 (Dentsply Maillefer, Ballaigues, Swiss) to approximately 5–6 mm depth in the gutta-percha. RB instruments 25/~ (VDW GmbH, Munich, Germany) activated with endodontic X-Smart Plus micromotor (Dentsply Maillefer, Ballaigues, Swiss) in “Reciproc All” setting was gently inserted into the pathway and pushed to remove the coronal part of gutta-percha. The instrument was then removed, and the material entrapped among the instrument spires (threads) was displaced using a sterile sponge. The RB was re-inserted with a gentle pressure in apical direction divided in 3–4 strokes, removed, cleaned from debris, and re-inserted. The absence of a plastic carrier allowed the subsequent penetration of RB #40 that was gently forced to the apex, avoiding excessive pressure on the root canal wall. Every 15–20 s the instrument was removed and cleaned from debris. The irrigation was made after plastic carrier removal at each step with a total amount of 3.0 mL of 5% NaOCl and 3.0 mL of 10% EDTA. Each canal was subjected to further irrigation with 1.0 mL NaOCl and 0.5 mL EDTA and final irrigation with 0.5 mL saline solution for 3 min. All the time necessary for instrumentation, irrigation and check of the instrument surface was digitally recorded, added, and reported as total working time. When the instrument reached the WL and no debris were no longer observed in between the spires, each canal was considered “clinically re-treated”, and the total working time reported. Finally, a radiograph of each retreated root was taken.

### 2.3. Retreatment Procedure with Manual K-File

An initial glide path was obtained as previously described. Each canal was shaped using K-File #30 to #40 with a modified crown-down technique. Irrigation was done after the plastic carrier removal, as previously described, and with the same time. A manual K-file instrument was extracted from the canal after gaining 3–4 mm and the material entrapped among the instrument spires (threads) was removed using a sterile sponge. When the #40 K-file reached the working length and no debris were observed in between the spires, the canal was considered “clinically re-treated”, and the final working time was reported. Finally, a radiograph of each root was taken.

### 2.4. Radiographic Evaluation of Root Canal Space Appearance

A periapical radiograph was used to measure the initial 2D area (in mm^2^) of root canal before any treatment (pre-operative area), after root canal treatment, and after secondary treatment. An open-source software (ImageJ, NIH software, Bethesda, MD, USA) was used for all the evaluations. Variation of root canal space appearance after the first treatment was measured and reported in percentages. The presence of radiopacity related to residual filling materials into the root canal after secondary treatment was assessed and measured in mm^2^.

### 2.5. ESEM-EDX Sample Preparation

Two longitudinal grooves were created in each root from apex to the coronal portion through a highspeed water-cooled handpiece with a diamond cylindrical bur. With a chisel placed above the canal, the root was divided into two longitudinal halves. One of the two halves was randomly selected and analyzed by ESEM-EDX [12] to detected root filling debris presence. ESEM analysis was done at different magnification (100× and 3000×) to observe macro and micro morphology of root canal dentin surface, location, and quantity of debris. The specimens were placed directly on the ESEM stub and examined without any previous preparation (uncoated samples). Operative parameters were low vacuum 100 Pascal, accelerating voltage of 20–25 kV, working distance 8.5 mm, and 133 eV resolution in Quadrant Back-Scattering Detector (QBSD) mode (0.5 wt% detection level, amplification time 100 μs, measuring time 60 s). 

EDX spectroscopy was used to identify and the element composition of dentin and debris/remnants. Qualitative and semiquantitative element (weight % and atomic %) content were investigated by applying the ZAF correction method, a procedure in which corrections for atomic number effect (Z), absorption (A) and fluorescence (F) are calculated separately. The presence of specific elements inside the composition of sealer or gutta-percha (i.e., Zr, W, Zn, etc.) was used as a trace element to confirm the remnant presence (i.e., sealer or gutta-percha, or dentin smear layer). Different dentine areas were detected using Image J software (NIH software, Bethesda, MD, USA). Dark-light grayscale quantification was used to isolate and measure different gray values areas. [13] Three measurements were done for each root canal third (total 9 for each sectioned root).

### 2.6. Statistical Analysis

Data were inserted in tables to be analyzed and were used to plot graphs. Differences between the two groups were analyzed using one-way ANOVA followed by RM Student–Newman–Keuls test (*p* value was set at 0.05).

## 3. Results

**Figure 2:**
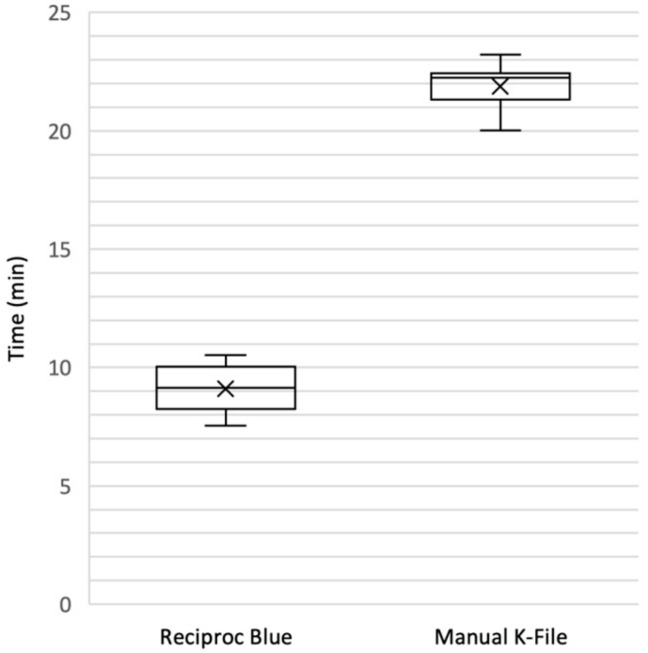
Retreatment working time. Manual technique required statistically significant more time than RB. In both techniques the clinical parameter to step the retreatment procedure was the absence of debris from instrument removed from root canals.

**Figure 3:**
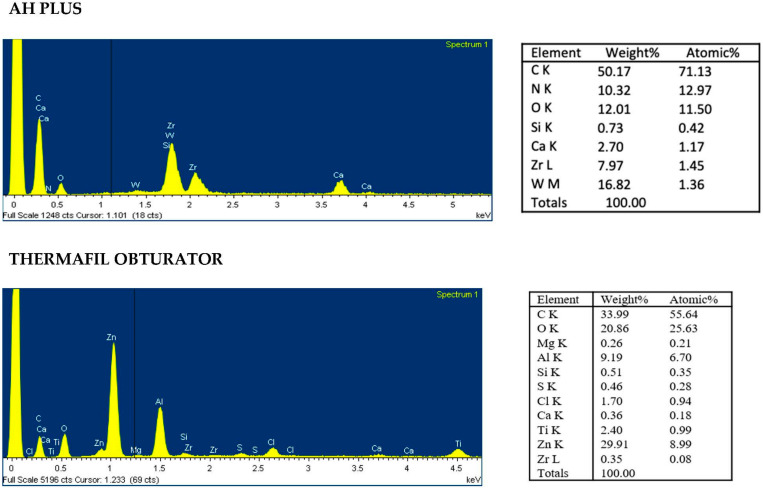
EDX spectra of sealer and carrier used in the present study.

**Figure 4:**
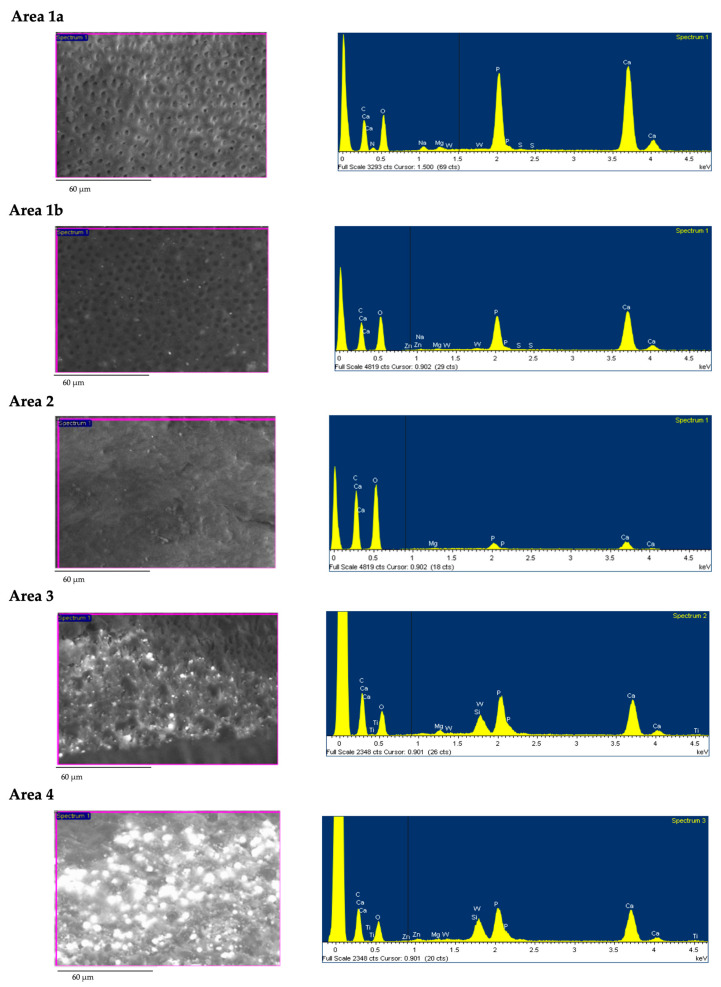
Dentine areas identified on root canal walls of secondary root canal treatments performed in the present study. The presence of specific elements inside the composition of sealer or gutta-percha (i.e., Zr, W, Zn etc.) was used as trace element to confirm the remnant presence (i.e., sealer or gutta-percha, or dentin smear layer).

**Figure 5:**
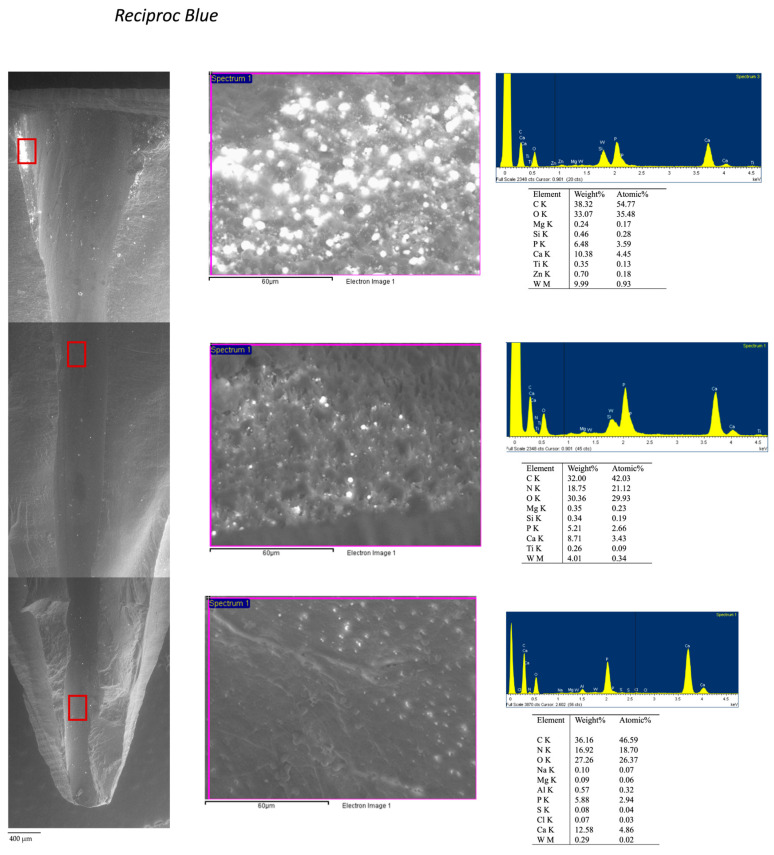
ESEM-EDX analysis at 100× magnification performed at the coronal medium and apical third of root retreated using a RB instrumentation. Coronal third revealed some areas filled with high electron dense remnants. Medium and apical third presented few debris, dentin smear layer (Ca and P) and some open and clean (no smear plugs) dentinal tubules. EDX analysis performed at 3000× magnifications on one area with electron dense remnants located at the coronal thread revealed a higher presence of Zn, Zr, and W, attributable to sealer and gutta-percha. Similarly, EDX at 3000× magnifications in one area located at the medium third with fewer electron dense debris revealed reduced presence of elements attributable to sealer (W). EDX at one area at the apical third revealed a high concentration of smear layer, with higher presence of Ca and P and low presence elements attributable to the sealers (W). Interestingly, traces of Ti were identified in correspondence of some areas with high electron dense remnants, which may be attributable to recurrent instrumentation (over instrumentation) of RB files in those sites.

**Figure 6:**
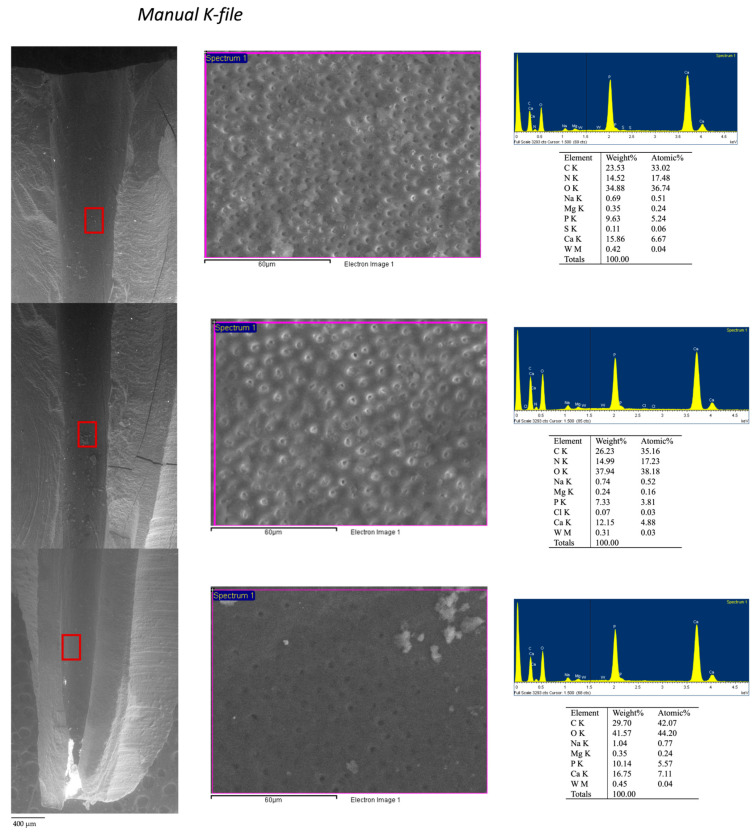
ESEM-EDX analysis at 100× magnifications performed at the coronal medium and apical third of root retreated using a manual instrumentation technique. Both coronal and medium third revealed few filling debris. Differently, apical third presented a higher presence of filling debris (evidenced as highly electron-dense deposits). EDX at 3000× on dentin areas free from filling deposits showed a limited presence of W, attributable to the sealer.

Results were summarized in Table 1, Table 2, Table 3, Table 4, Table 5 and Table 6 and Figure 2, Figure 3, Figure 4, Figure 5 and Figure 6.

### 3.1. Radiographic Measurement of Root Canal Area

Pre-operative root canal area was similar in both groups (Table 1). After the first root canal treatment, the area increased in both RB and Manual K-File. No differences were observed between RB and manual K-file groups (*p* > 0.05). Statistically, differences were observed among the pre-treatment, treatment, and re-treatment area for both groups, the re-treatment area was significantly higher than the pre-operative area (*p* < 0.05). Manual K-file technique was responsible for greater instrumented area after re-treatment procedure, although not statistically significant (*p* > 0.05).

### 3.2. Working Time and Residual Radiopacity

A statistically significant difference was reported between the re-treatment working time of the two groups (Table 2). The working time of manual K-file was statistically higher than the RB technique.

RB group removed filling material mostly in blocks. K-file removed plastic carrier in small fragments with a small piece of gutta-percha as observable under optical magnifications. Several K-Files underwent irreversible deformation (and substituted) during retreatment because of the difficulty of penetration toward the apex region.

### 3.3. Radiographic Evaluation 

Radiographic presence of filling material detectable in root canals after re-treatment procedures is reported in Table 3. The values were not statistically significant (*p* < 0.05), although the RB group was responsible for a lower presence of radiopaque remnants (mean value was 2.59 ± 1.94).

### 3.4. ESEM-EDX Analysis of Dentin Area

Root dentin surface was carefully inspected under ESEM at coronal, medium, and apical thirds of the canal at 100× and 3000× and then re-evaluated with EDX to obtain three spectra of each coronal, medium and apical root regions. 

Based on ESEM morphology and on EDX element composition, dentin surface was schematized in four different dentine Areas (Area 1–4; see Table 4). Morphological aspects (dentine open tubules or plugged with debris, smear layer, etc.) were used to identify the areas. Debris and remnants frequently spread on the dentin surface were identified thanks to their chemical composition (used as tracer elements) by the use of the EDX. So, for example, the presence of W or Zr was in relation with the presence of sealer debris (AH Plus). Representative EDX spectra of set AH Plus sealer and Thermafil obturator is reported in Figure 3.

Area 1 identifies a sound smooth instrumented dentin free from any smear layer. All dentinal tubules proved open and clean (no smear plugs/sealer debris inside). This area was subdivided into two subgroups, as EDX investigation revealed differences in the surface chemical compositions of intratubular dentin substrate. In Area 1a, sound dentine surface proved the typical chemical composition of dentinal apatite mainly composed by Ca and P (both attributable to the inorganic composition of apatite) and N (from dentin organic components, i.e., collagen). Area 1b revealed a similar ESEM morphology, but EDX spectra revealed no traces of N, the trace element of collagen.

Area 2 identifies a smooth dentin surface completely covered by a thin homogeneous smear layer that completely obscured the dentinal tubules filled by smear plugs. EDX revealed the absence of N peaks and high presence of Ca, P peaks. The smear layer was collagen-free, as probably the resultant of NaOCl.

Area 3 was defined as a dentin surface covered by many filling components immersed into a thick smear layer with no tubules exposed. ESEM investigation identified high electron-dense globular and spread deposits mixed in an irregular smear layer. EDX microanalysis revealed the presence of consistent peaks of Zr, W, and Zn (all elements attributable to filling materials debris), low presence of Ca and P peaks, and no N. Smear layer proved completely depleted by N.

Area 4 defined a dentin surface heavily contaminated by remnants of filling materials. These areas were identified at ESEM observation as irregular areas with much electron dense debris of 10–300 micron mixed with the thick and multilayered smear layer. EDX microanalysis revealed Zr, W, Si, and Zn peaks all elements of sealer and gutta-percha. Limited Ca, P, and no N peaks (relative to dentine components) were detected, suggesting dentin surface almost totally filled by artificial debris contaminated smear layer.

The four dentin areas identified for K-file and RB groups are reported in Figure 4 and in Table 5, Table 6. No statistically differences were observed between the two groups. Figure 5; Figure 6 report the analysis and schematic representation with coronal, medium and apical appearance of dentin surface and relative EDX spectra.

RB: Debris was observed at 100× magnification, the inner dentin surface presented a uniform morphology with a modest number of grooves. Differences were observed at the coronal medium and apical portion of retreated roots. The coronal third revealed few areas filled with high electron dense remnants. Analysis of these areas at 3000× magnification revealed the typical morphology of artificial smear layer mixed with filling material: fine granules occluded dentinal tubules and contained W and Si and the other sealer components. Rare dentinal tubules were visible most of which filled by sealer (W presence). The medium third presented little debris, dentin smear layer (Ca and P), and some open and clean (no smear plugs) dentinal tubules. The apical third presented few filling debris, but many dentinal tubules still were filled with sealer and smear layer, as confirmed by W, Zn, and Si (Figure 5). 

Manual K-file: at low magnification (100×) morphology of inner dentine surface was uniform well detectable and identifiable. Coronal third was characterized by the presence of great number of open dentinal tubules and absence of smear layer. At 3000× magnification, some debris covered limited portion of dentin surface in the areas. EDX revealed in these areas, traces W and Zn, attributable to root canal filling materials. Medium third presented many open dentinal tubules and uniform surface. Apical third showed dentin smear layer and rare dentinal tubules with few filling debris, as confirmed by W and Zn detected by EDX (Figure 6).

## 4. Discussion

This study aimed to explore the composition and the morphology of debris inside root canals after secondary root treatment of teeth filled with AH Plus and Thermafil system. The choice of this obturation system was made as it is widely used in endodontics, is predictable, non-time consuming, and with a fast learning curve [15]. Limited information is present in literature regarding the morphology of the debris, the root canal wall morphology, and microchemical modifications after secondary root canal treatment, most of the studies focused on root canal filling materials removal efficiency through 3D radiographic analyses [11,16,17,18,19].

The combined use of ESEM and EDX offered new information on the morphology and disposition of remnants inside the root canal and their interaction with the smear layer. The position and number of remnants were placed in relation with debris finding on radiographies. Periapical radiographs still represent the only clinical diagnostic images of the presence/absence of residual filling materials during the entire operative procedure and therefore, the only clinical information available for the clinician during the endodontic therapy.

ESEM offers the possibility to directly observe the morphology of root inner dentin surface after re-treatment without the critical dry preparation and sample artifacts [12]. The use of EDX combined with ESEM identified the chemical elements included in the composition of gutta-percha and sealer debris. If trace elements were still present on the dentin surface, they were index of contaminated debris still deposited into the canal after retreatment. The presence of remnants indicates the incompleteness of secondary root treatment.

The study aimed as one of the main purposes to correlate the radiographic (clinical) findings with the surface morphology and composition analyzed by ESEM-EDX (laboratory) and produced by two techniques (RB and conventional manual k-file technique). To the best of our knowledge, no information is present on the use of the RB technique for secondary root canal treatment procedures. The EDX analysis of the dentin surface differentiated the debris traces elements from AH Plus and gutta-percha (from Thermafil). EDX also offered the possibility to detect the composition of root dentin after irrigation procedures and their modification with respect to sound dentin [20,21].

Many samples proved Area 1a and Area 1b with smooth dentin surface and open dentinal tubules in a large portion of the root canal (60–70% of root canal dentin surface) demonstrating the efficacy of both instrumentation techniques in removing filling materials. Sound dentin composition was confirmed by the presence of Ca, P, and N peaks in the composition.

Area 2 was characterized by the presence of a smear layer and occupied approximately 14–24% of the dentin surface in both sample groups. The smear layer is usually produced by instrument motion [20,22,23] and it is composed by Ca, P, and N, all elements of dentine [20,24,25].

Interestingly, both Area 1b and Area 2 was found to be completely depleted by N and consequently free from the organic components. Collagen depletion was caused by the use of NaOCl as irrigant solution in Area 1b and by incomplete mineral smear layer removal in Area 2. In dentin Area 2 it was evident that NaOCl was able to remove collagen fibrils (and N) from the smear layer but unable to remove the smear layer [23,25,26]. EDTA, a chelating agent, acted with great efficacy in surface dentin corresponding to Area 1 and it was able to dissolve the entire thickness of the smear layer and so exposed sound healthy dentin, as confirmed by previous studies [20,25].

The smear layer of Area 2 and dentin surface of Area 1b were greatly depleted by collagen and completely free from any organic components, as no N peaks were evident. N element is part of the amide functional groups of the collagen organic structure [27].

Debris was clearly detectable by ESEM in large portions of the root dentin wall and identified as Areas 3 and 4. Filling debris were “spread” and “smashed” on the dentin surface and usually packed and mixed with the smear layer produced by endodontic instruments. Areas 3 and 4 were characterized by a complex smear layer substrate. Area 3 presented many filling debris—confirmed by Zn, W, Zr, and Si peaks—embedded in a collagen-free smear layer, with the identification of only Ca and P peaks. Filling and dentin debris produced a sort of new artificial smear layer mainly observable at apical third (Area 4), which almost corresponds to the radiopaque filling material identified in periapical radiographs. The artificial smear layer was extremely thick as it was able to mask the Ca, P, and N peaks of EDX spectra, showing lowered values than sound dentine. The amounts of remnants occupied approximately 10–15% of root canal surface in both groups and were more frequent at apical third. The fragmentation of sealer-gutta-percha was operated by instrument and confirmed by the diffuse presence of remnants [28].

Areas 3 and 4 may correspond to the areas previously described by Rodig et al. [16] using Micro-CT. Therefore, the presence of radiopacities visible at apical thirds may guide the clinician to consider the presence of many remnants into the canal and the necessity to remove them. The ESEM analysis of the dentin surface created a map of different areas that reflects the effectiveness of instrument action and irrigation effect on dentin. The element map distribution and the morphology of debris were similar in both groups.

AH Plus sealer is able to bond to the dentin surface [24,29]. It is flowable and able to expand and seal after a short time [30]. RB and K-file movements created many fragmentation and chops into the thickness of sealer/gutta-percha structures. Debris fragments were entrapped into the spires and removed when the instrument is out from the canal. The morphology of some fragments embedded on the smear layer supports the concept of spreading on dentin walls. Other remnants from AH Plus (W and Zr element spectra) were concentrated inside dentinal tubules of apical thirds, probably consequence of capacity to bond dentin [24,29] and to deeply penetrate into the tubules [31].

Radiographies made after secondary root treatment demonstrated that the area of the root canal was approximately 30% greater than the pre-operatives, as the results of additional dentin removal after instrumentation. No differences in the radiographic area were observed between the two groups. So, both treatments were unable to completely remove debris from the root canal, as confirmed by ESEM-EDX and Rx. Strategies to remove the smear layer may be to alternate irrigating solutions (namely EDTA and NaOCl) and to increase root canal preparation with instruments with larger diameters. However, larger root canal preparation may compromise root integrity, causing fracture and tooth extraction [4]. In the present study, irrigation was performed following a protocol currently used in clinical practice and reported in several clinical studies [15,32]. Ultrasonic activation of irrigating solutions into the root canal showed a minimal improvement but did not provide complete removal of root canal filling materials [33,34].

Many recent investigations performed with micro-CT technique but different NiTi instruments confirmed that the different retreatment technique is not able to completely remove debris of filling materials [8,17,18]. The results of the present study are in the same directions of previous investigations made with micro-CT and other methods on different NiTi instruments [10,19,35,36].

The optical absence of any debris into the threads of instruments removed from the root canal was selected as a main clinical index to stop the retreatment procedures. Other clinical parameters were used to stop the retreatment procedures, such as the time to reach the apex [16,37]. 

RB technique revealed statistically lower working time than the manual procedure to remove most of the packed filling materials. No instrument fractures were reported for this instrument.

Azim et al. [19] reported that WaveOne, a reciprocating system, showed a large amount of gutta-percha and apical extrusion that was lower than other instrumentation.

The quality of the endodontic treatments is constantly improving [37] but the outcome of the endodontic treated teeth is not yet 100% successful [15,32,38]. Again, the number of secondary root canal treatments is expected to increase in the near future, so it is extremely important to have safer and simpler instruments and irrigation procedures to use in complex root canal treatments.

Limitation of the present study may be that all teeth were single-root and treated after crown removal, the evaluated parameters need to be validated also on curved and multiple-root teeth. The present ESEM-EDX protocol could be useful to assess whether collagen depletion occurs with other irrigant solutions or other concentration.

## 5. Conclusions

The study offered new information and reported conditions crucial for secondary root canal treatment outcome:

Both k-manual and RB were able to remove great part of Thermafil and AH Plus fillings, as confirmed by a great portion of dentin in Areas 1 and 2. RB was safely used for a single-straight root canal retreatment filled with Thermafil and AH Plus. RB required less time to complete a secondary root canal treatment than K-file manual instrumentation.

ESEM-EDX analysis detected a great number of filling tracer elements (W, Zr, Zn, and Si) embedded in the thickness of the smear layer produced by tested techniques. The presence of these remnants may be related to the persistence of other remnants or bacteria into the root canal.

EDX findings support the concept that the dentin surface is strongly modified by combined used of instruments and irrigant solutions (NaOCl and EDTA). Both solutions created a dentine surface depleted from collagen-organic components.

## Figures and Tables

**Table 1:** Pre-operative root canal area (mean ± standard deviation (SD); mm^2^), after root treatment and after secondary root treatment. No statistical differences were observed. NS = no significant differences between groups (*p* > 0.05)

Root Canal Area	Reciproc Blue (*n* = 12)	Manual K-File (*n* = 12)
Pre-operative area (mm^2^)	7.01 ± 1.96 NS	6.93 ± 1.72
Treatment area (mm^2^)	9.68 ± 2.23 NS	8.42 ± 1.61
Re-treatment area (mm^2^)	10.49 ± 1.50 NS	10.95 ± 2.61

**Table 2:** Working time (min, expressed as mean ± SD).

Reciproc Blue (*n* = 12)	9.10 ± 1.02 *p* < 0.0001
Manual K-File (*n* = 12)	21.54 ± 4.46

**Table 3:** Radiopaque remaining filling materials (remnants) visible in periapical radiographies and expressed as area with residual radiopacity (mm2; mean ± SD).

Reciproc Blue (*n* = 12)	2.59 ± 1.94 NS
Manual K-File (*n* = 12)	3.18 ± 2.61

**Table 4:** Definition of dentine areas.

Type of Dentin Areas	Definition	ESEM	EDX
Area 1a	Clean, smooth, regular and sound instrumented dentine. No grooves and pits observed.	100×: Absence of high electron dense areas. 3000×: Dentinal tubules well visible with no high electron dense remnants and no smear layer. Absence of debris and remnants	Presence of Ca, P and N elements with apatite Ca/P ratio and dentin Ca/N ratio. No/limited presence of sealer and gutta-percha tracer elements (Zr, Zn, Si, W, Ti on dentin surface).
Area 1b	Instrumented dentine with signs of deprotenized dentin. No smear layer	100×: Presence of low high electron dense debris. 3000×: Dentinal tubules partially covered with smear layer	Presence of Ca and P. Absence of N. Absence (or low presence) of tracer elements of sealer and gutta-percha (Zr, Zn Si, W).
Area 2	Smooth dentin surface covered by a collagen-free deprotenized smear layer	100×: few high electron dense debris. 3000×: Dentinal tubules covered by a thin smear layer with free from any remnants and electron dense particles	Presence of Ca and P. Absence of N. No/rare presence of sealer and gutta-percha tracer elements (Zr, Zn, Si, W).
Area 3	Contaminated smear layer containing debris from filling materials. Absence of collagen	100×: widespread electron dense deposits and presence of remnants. 3000×: Many remnants and materials spread or immersed into thick smear layer with only sporadic dentinal tubules noticeable	Moderate presence of Ca and P elements Absence of N Moderate presence of sealer and more gutta-percha tracer elements (Zr, Zn, Si, W).
Area 4	Heavy contaminated dentin surface for presence of remnants and debris of filling material. Irregular surface	100×: uniform and compact high electron dense deposits. 3000×: All surface covered by spread remnants Dentinal tubules completely masks by uniform and electron dense layer. Debris and remnants visible of dentine.	Reduced presence of P and Ca and N. High presence of sealer and gutta-percha tracer elements (Zr, Zn, Si, W).

**Table 5:** Root canal areas identified by ESEM-EDX and expressed as percentages (mean ± SD) of retreated teeth using manual K-file instruments.

Manual	Coronal	Medium	Apical
Area 1	69.0 ± 26.1	60.0 ± 7.1	65.5 ± 4.9
Area 2	18.0 ± 7.7	24.5 ± 0.7	24.1 ± 1.4
Area 3	6.9 ± 5.9	12.0 ± 4.3	5.5 ± 3.5
Area 4	5.5 ± 4.8	4.1 ± 2.4	8.5 ± 0.7
Total	100.0	100.0	100.0

**Table 6:** Root canal areas expressed as percentages (mean ± SD) of analyzed teeth using Reciproc Blue instrumentation.

Reciproc Blue	Coronal	Medium	Apical
Area 1	69.0 ± 4.2	81.0 ± 8.4	72.5 ± 10.5
Area 2	19.0 ± 7.1	14.0 ±5.2	16.5 ± 12.4
Area 3	4.5 ± 0.7	2.5 ± 0.6	6.0 ± 0.3
Area 4	6.5 ± 0.7	2.5 ± 0.5	5.0 ± 0.2
Total	100.0	100.0	100.0
